# Healthy Lifestyle in Adolescence: Associations with Stress, Self-Esteem and the Roles of School Violence

**DOI:** 10.3390/healthcare12010063

**Published:** 2023-12-27

**Authors:** Alba González Moreno, María del Mar Molero Jurado

**Affiliations:** Department of Psychology, University of Almería, 04120 Almería, Spain

**Keywords:** healthy living, stress, self-esteem, school violence, adolescence

## Abstract

A healthy lifestyle is considered by young people as the adoption of positive behaviors, such as a balanced diet, regular exercise, or the decreased consumption of harmful substances. Living a healthy lifestyle during adolescence promotes a better quality of life and psychological well-being in adulthood. The objective of this research is to identify how a healthy lifestyle is related to stress, self-esteem, and school violence roles. The sample is composed of a total of 743 adolescents aged between 14 and 19 years. The instruments used were the Healthy Lifestyles Questionnaire (CEVS-II), the Student Stress Inventory (SSI-SM), the Rosenberg Self-Esteem Scale (RSES), and an ad hoc questionnaire to evaluate school violence roles. The results obtained indicate that there are negative correlations between healthy lifestyle and stress, but positive correlations between healthy lifestyle and self-esteem. Adolescents who participate in situations of school violence have a higher mean of substance use and stress. However, those who do not experience such situations have higher mean self-esteem and lead a healthy lifestyle. In addition, the fact of suffering stress increases the probability of being a victim or an aggressor. Living a healthy lifestyle can have significant implications for health promotion and positive adolescent development.

## 1. Introduction

The World Health Organization defines lifestyle as a general way of living that arises from the interaction between living conditions in a broad sense and individual behavior patterns influenced by sociocultural factors and personal traits [1]. The same organization states that adolescence is a transitional stage between childhood and adulthood, spanning from 10 to 19 years old [2]. Adolescence is crucial for future health and disease, as habits acquired during this period may persist into adulthood, such as alcohol consumption, healthy food intake, and the development of chronic diseases [3]. During this period, adolescents are exposed to various influences that can affect their lifestyle and overall well-being [4]. Healthy lifestyle in adolescence has been the subject of increasing interest, as it has been shown to be associated with several positive outcomes, such as improved quality of life, reduced risk of chronic diseases, and increased psychological well-being [5,6,7].

Recent studies show that adolescents perceive healthy lifestyle as having a balanced diet, regular physical activity, disease prevention, and positive attitudes, such as eating fruits and vegetables, and negative attitudes, such as skipping meals and eating junk food [8]. This same research indicates that the home environment, school campus, peers, and concern for the quality of fast food had positive influences, while academic schedules, campus restaurants, and the sociocultural environment were negative influences towards a healthy lifestyle, with computer and Internet activity being the main sedentary activity. Self-efficacy and health literacy are estimated to be determinants of healthy lifestyle behaviors in young people [9]. Despite having awareness of what it means to lead a healthy lifestyle, previous literature establishes that adolescents do not tend to carry positive healthy attitudes, with such healthy behaviors being lower as age advances [10].

Healthy lifestyle encompasses a variety of dimensions, such as balanced diet, regular physical activity, adequate sleep, and the avoidance of risky behaviors, such as excessive screen use, among others [11,12,13]. However, the current scientific literature on how factors such as stress, self-esteem and violence roles relate to a healthy lifestyle in adolescence is scarce.

During adolescence, young people face a few challenges that can generate stress, such as finding their identity, adapting to the expectations of school, family and friends, and making decisions related to their future [14]. These factors can influence adolescents’ ability to adopt a healthy lifestyle, as stressful situations can lead to unhealthy behaviors, such as poor diet or lack of physical activity [15,16,17]. All of this may be conditioned by the fact that the use of social networks in adolescence is linked to the purchase of harmful substances [18]. In addition, experiencing stressful situations increases the probability of consuming higher levels of harmful substances, such as alcohol or tobacco [19]. On the other hand, young people who experience exposure to school violence, such as victims and aggressors, show a lower ability to cope with stressful situations [20]. The same occurs with cyberbullying, since young people who suffer cyberbullying present higher levels of anxiety and depression [21]. This negative association is also represented in self-esteem, because young people who present high levels of stress tend to have lower self-esteem [22]. Therefore, studies suggest that positive psychosocial factors, such as self-esteem, may have a protective effect against negative psychosocial factors, such as stress in adolescents [23].

Self-esteem refers both to one’s own subjective evaluation of personal worth and to the feedback obtained from the interpretations of other subjects [24]. During adolescence, one of the most relevant factors that promote psychological well-being is self-esteem [25]. Moreover, self-esteem is estimated to influence the overall health of individuals, both in adolescence and adulthood [26]. Low self-esteem can negatively affect adolescents’ motivation to take care of their health and adopt healthy habits [27]. Conversely, high self-esteem may be associated with a higher likelihood of engaging in healthy behaviors, such as good nutrition and regular exercise [28]. In relation to school violence, it is observed that individuals with higher self-esteem present a lower susceptibility to being victims of bullying and/or cyberbullying [29].

The roles of violence can play a significant role in the adoption of a healthy lifestyle in adolescence. Adolescents who are exposed to violence, in this case within the school setting, face difficulties in maintaining healthy habits and tend to be at high risk for risky behaviors, such as substance use, physical inactivity, and an unhealthy diet [30,31]. Peer victimization can negatively affect young people’s life satisfaction, decreasing their perception of community support and impairing their development and quality of life [32]. In addition, positive correlations have been found between cybervictimization and levels of anxiety, depression, and stress, with adolescent girls having higher means [33]. Young people who lead a healthy lifestyle based on good hygiene, physical activity, and abstinence from tobacco have a lower probability of being bullied or being victims of school violence [34].

### Objective and Hypothesis of the Study

Despite the importance of healthy lifestyle in adolescence, there is still a gap in the understanding of the associations between stress, self-esteem, violence roles, and the adoption of healthy habits. Therefore, the aim of this study is to identify how a healthy lifestyle relates to stress, self-esteem, and school violence roles. The initial hypotheses considered are the following:

**H1:**  *A healthy lifestyle is positively related to self-esteem and negatively related to stress*.

**H2:**  *There are differences according to the role of violence (victim and aggressor) in healthy lifestyle, stress, and self-esteem*.

**H3:**  *There are sex differences between adolescent victims and perpetrators in healthy lifestyle, stress, and self-esteem*.

**H4:**  *The different self-esteem groups (low, medium, and high) show differences in healthy lifestyle and stress*.

**H5:**  *Healthy lifestyle and stress are predictors of being a victim or an offender in adolescence*.

## 2. Materials and Methods

### 2.1. Study Design and Participants

This quantitative study with a descriptive cross-sectional design followed the guidelines established by the STROBE statement for carrying out cross-sectional studies and guaranteeing the reliability of the research [35]. The sample consisted of 743 young people aged 14–19 years (*M* = 14.99; *SD* = 0.86). A total of 50.7% were female (*n* = 377) and 49.3% were male (*n* = 366). All the participants were students from public secondary schools in the province of Almería (Spain). Of the students, 50.7% were in their third academic year, while 49.1% were in their fourth year. Most of the adolescents were of Spanish nationality (92.9%), although students of other nationalities also participated.

### 2.2. Instruments

The measurement of the different variables in adolescents was carried out by means of a booklet containing a series of ad hoc questions and validated instruments. The variables of the present study were evaluated as follows:

- Healthy lifestyle. This variable was assessed by means of the Healthy Lifestyle Questionnaire (CEVS-II) validated in the Spanish population [36]. Said questionnaire contained 27 items grouped into seven dimensions, whose internal consistency was good in each of its dimensions: healthy diet (*α* = 0.59), respect for mealtimes (*α* = 0.65), rest habits (*α* = 0.78), tobacco consumption (*α* = 0.89), alcohol consumption (*α* = 0.86), consumption of other drugs (*α* = 0.81), and physical activity (*α* = 0.82). The entire instrument is answered using a five-point Likert scale ranging from 1 (strongly disagree) to 5 (strongly agree).
- Stress. The level of stress experienced by the participants was assessed using the Spanish adaptation of the Student Stress Inventory (SSI-SM) [37,38]. The instrument has 22 items that are answered using a five-point Likert-type scale (1 = never; 2 = rarely; 3 = sometimes; 4 = often; 5 = a lot). This scale provides a total stress score and a score for three dimensions of stress: emotional manifestations, physiological manifestations, and behavioral manifestations. The internal consistency was good for the total scale (*α* = 0.89) and the measure of emotional manifestations (*α* = 0.87), acceptable for physiological manifestations (*α* = 0.71), and hypothetical for behavioral manifestations (*α* = 0.65).
- Self-esteem. To examine self-esteem, the original Rosenberg Self-Esteem Scale (RSES) was used in its Spanish version adapted for adolescents [39,40]. This instrument is made up of 10 items focused on knowing the feelings that a person has about him/herself and which are answered using a Likert-type scale with four response options (1 = strongly disagree; 2 = disagree; 3 = agree; 4 = strongly agree). It presents a total self-esteem score that can be interpreted in two ways: low self-esteem (less than 25 points) and high self-esteem (30 to 40 points). The internal consistency obtained in this instrument was good (*α* = 0.83).
- Roles of School Violence. The students’ perception of school violence has been examined by means of questions incorporated in the ad hoc. These questions are answered dichotomously and are focused on two roles: victim (e.g., “Have you suffered episodes of violence from your classmates?”) and aggressor (e.g., “Have you exercised violence on your classmates?”).

### 2.3. Procedure and Data Collection

Once the instruments had been selected and the data collection booklet had been prepared, we contacted several secondary schools in different municipalities in the province of Almería (Spain). A total of six schools agreed to participate in the study, so an appointment was made with the school management to attend one day and have the students fill out the booklets themselves. Before data collection began, all students and their legal guardians were informed of the purpose of the research and gave their consent to participate in the investigation. This collection was conducted from February to June 2022. This study was approved by the Bioethics Committee on Human Research of the University of Almeria with reference UALBIO2021/025.

### 2.4. Data Analysis

Data analysis was performed using SPSS statistical software version 28 [41]. Cronbach’s alpha coefficient was used to test the reliability of the instruments used. This coefficient is interpreted as follows: <0.5 unacceptable, >0.5 poor, >0.6 questionable, >0.7 acceptable, >0.8 good, and >0.9 excellent [42].

A descriptive analysis was performed to provide relevant information about the students who participated in this study. In addition, a Pearson bivariate correlation analysis was performed to determine if there was a relationship between the variables studied. The absolute values obtained were interpreted according to the following categories: no correlation between 0 and 0.10; weak correlation between 0.10 and 0.29; moderate correlation between 0.30 and 0.50; strong correlation between 0.50 and 1.00 [43].

In addition, Student’s *t*-test for independent samples was performed. This test was performed to examine the differences between the profiles of violence (victims and aggressors) in the variables analyzed and their corresponding dimensions. Cohen’s *d* [44] was also calculated to estimate the effect sizes: small 0.50; medium 0.50–0.80; large ≥0.80.

To determine the differences according to self-esteem groups (g1 = low self-esteem; g2 = medium self-esteem; g3 = high self-esteem) in comparison with the different dimensions of a healthy lifestyle and stress, an analysis of variance (ANOVA) was performed. To know the effect size in such analysis, the eta squared (*η*^2^) was used, where around 0.01 is usually considered to be a small effect, around 0.06 indicates a moderate effect, and above 0.14 is considered a large effect [45].

Finally, a binary logistic regression analysis was performed to determine which dimensions of healthy lifestyle, stress, and self-esteem act as risk or protective factors for being a victim or an aggressor in adolescence. The probability has been assessed using the odds ratio value (exp (ß) as a measure of the effect size [46]. Statistical significance was set at a *p* value of less than 0.05.

## 3. Results

### 3.1. Descriptive Analyses and Correlations

The results obtained show, in Table 1, the correlations between the variables analyzed.

In reference to healthy lifestyle and stress, we can see how all the dimensions of stress (emotional, physiological, behavioral, and total stress manifestations) correlate negatively with healthy diet, respect for mealtimes, rest habits, and physical activity. However, these dimensions of stress are positively correlated with the dimensions related to consumption (consumption of tobacco, alcohol, and other drugs).

As for the associations between healthy lifestyle and self-esteem, something like that mentioned above can be observed. Self-esteem correlates positively with healthy diet, respect for mealtimes, rest habits, and physical activity. However, the association is negative with the dimensions related to consumption.

Regarding self-esteem and stress, it was found that there is a significant negative relationship between self-esteem and the different dimensions of stress (emotional, physiological, behavioral, and total stress manifestations).

The correlation between the age of the participants and the variables examined was tested. The statistical results indicate that there is a significant relationship between age and the different dimensions of healthy lifestyle (healthy diet, respecting meal times, resting habits, tobacco use, alcohol consumption, use of other drugs, and physical activity). However, no significant relationships were found between age and either stress or self-esteem.

### 3.2. Differences between Victims and Aggressors: Healthy Lifestyle, Stress, and Self-Esteem

The data in Table 2 show the differences between two profiles of violence: victims and aggressors.

Adolescents who perceive themselves as victims have higher scores in the use of other drugs and in the different dimensions of stress (emotional, physiological, behavioral, and total stress). However, nonvictimized youth show higher rest habits, physical activity, and self-esteem.

In reference to aggressors, they stand out for tobacco and other drug use, physical activity, and higher levels of total stress. On the other hand, nonoffenders score higher in healthy diet, respect for mealtimes, rest habits, and self-esteem.

Having examined the differences according to the profile of violence, we wanted to investigate the differences according to sex between young people who perceive themselves as victims and aggressors.

Table 3 shows that girls who are victims show greater emotional manifestations of stress. In contrast, boys who are victims tend to be more physically active, show greater behavioral manifestations of stress, and have higher self-esteem.

As for young people who self-perceive themselves as aggressors, no significant differences were obtained.

### 3.3. Differences between Different Self-Esteem Groups with a Healthy Lifestyle and Stress

In view of the differences according to the profile of violence examined above, the aim was to investigate whether there are also differences according to the level of self-esteem in young people.

Table 4 identifies, through an analysis of variance (ANOVA), that there are differences according to the level of self-esteem. The group of adolescents with a higher level of self-esteem (g3) scored significantly higher in most of the healthy lifestyle dimensions: healthy diet (*F* = 18.11; *p* < 0.001; *η*^2^ = 0.04), respect for meal times (*F* = 28.10; *p* < 0.001; *η*^2^ = 0.07, rest habits (*F* = 37.14, *p* < 0.001; *η*^2^ = 0.09), alcohol consumption (*F* = 6.49, *p* < 0.002; *η*^2^ = 0.01), consumption of other drugs (*F* = 7.48, *p* < 0.001; *η*^2^ = 0.02), and physical activity (*F* = 16.80, *p* < 0.001; *η*^2^ = 0.04). The only dimension referring to healthy lifestyle in which no significant differences were found between the different groups was tobacco consumption.

In relation to stress, the results show that the group of young people with higher self-esteem (g3) had lower mean scores in the stress dimensions: emotional manifestations (*F* = 145.17; *p* < 0.001; *η*^2^ = 0.28), physiological manifestations (*F* = 77.77; *p* < 0.001; *η*^2^ = 0.17), behavioral manifestations (*F* = 36.31; *p* < 0.001; *η*^2^ = 0.08), and total stress score (*F* = 134.104; *p* < 0.001; *η*^2^ = 0.26).

### 3.4. Probability of Being a Victim and Being an Aggressor in Adolescence: Relationships According to Healthy Lifestyle, Stress, and Self-Esteem

A binary logistic regression analysis was performed to determine the probability between the dependent variable (being a victim/being a perpetrator) and the independent or predictor variables (healthy lifestyle, stress, and self-esteem).

As can be seen in Table 5, this analysis has been carried out in two different ways: one for being a victim and the other for being an aggressor.

In terms of the probability of being a victim of school violence, it is estimated that physiological manifestations of stress are a risk factor for being a victim. In statistical terms, the odds ratio (OR) shows that adolescents with physiological manifestations of stress have a 1.07 higher chance of being a victim of violence.

On the contrary, for being an aggressor, it has been obtained that behavioral manifestations of stress can be a risk factor. Following the statistical results, the odds ratio (OR) indicates that young people who present behavioral manifestations of stress have a 1.16 higher chance of showing aggressive attitudes.

## 4. Discussion

This research consisted of finding out how adolescents’ healthy lifestyles are related to stress, self-esteem, and the roles involved in school violence.

Adolescence is a crucial stage in the health of young people because the behaviors acquired at this age have a great influence on later ages [3,4]. We wanted to investigate this vital period because of the positive aspects of leading a healthy lifestyle in adolescence [5,6,7]. Leading a healthy lifestyle is associated with the promotion of certain positive behaviors, such as engaging in regular physical activity, eating a balanced diet, or reducing the consumption of harmful substances or screens [8,11,12,13]. It is estimated that young people do not lead a healthy lifestyle, whereas possessing self-efficacy and health literacy is a determinant for adolescents to carry out these types of positive health behaviors [9,10].

The results obtained indicate that there is a negative relationship between stress and certain healthy lifestyle behaviors (healthy diet, respect for mealtimes, rest habits, and physical activity). However, this association is positive between self-esteem and these positive health behaviors. Thus, we can indicate that hypothesis 1 of our study is fulfilled. This idea is supported by previous studies because stressful situations can lead to unhealthy behaviors, such as lack of physical activity or consumption of harmful substances [15,16,17], whereas self-esteem is associated with healthy behaviors [27,28].

These differences in healthy lifestyle are also associated with school violence roles. The data analyzed show that there are significant differences between healthy lifestyle habits, stress, and self-esteem according to the profile of violence, thus confirming hypothesis 2. These data show that adolescents who have suffered episodes of school violence (victims) or who have exercised violence to their peers (aggressors) show a higher tendency in stress and consumption of harmful substances. However, adolescents who have not participated in situations of school violence (nonvictims/nonaggressors) have higher self-esteem and healthy behaviors. These results may be associated with youth who experience school violence situations having lower ability to cope with stress [20], which is related to a higher consumption of harmful substances and a lower self-esteem [19,22]. However, higher self-esteem is related to a lower probability of being a victim of school violence [29]. Studies address that youth exposed to violence have risk behaviors, such as substance abuse and worse life satisfaction [30,31,32]. In addition, adolescents who lead a healthy lifestyle are less likely to be involved in school violence [34]. Attending to hypothesis 3, this has also been accepted, since sex differences were found between girls and boys who are victims of school violence. The data reflect that girl victims have greater emotional manifestations of stress, compared to boy victims who have more behavioral manifestations, physical activity, and self-esteem. As stated by Molero et al. [33], girls have higher mean scores in stress, anxiety, and depression.

The fact of having a higher or lower level of self-esteem on the part of adolescents has also been evaluated. The analysis of variance has confirmed hypothesis 4 of this study, which refers to the existence of differences according to the level of self-esteem in healthy lifestyle and stress. The data show that the group of adolescents with higher self-esteem show better health behaviors and less stress. Previous literature indicates that self-esteem influences both psychological well-being and general health of individuals [25,26], acting as a protective factor against negative psychosocial constructs, such as self-esteem [23]. On the other hand, attending to hypothesis 5, it can be indicated that it has been half-accepted, since significant scores have been found in stress but not in healthy lifestyle. Thus, these data indicate that young people who present manifestations of stress are more likely to be involved in situations of school violence as a victim or an aggressor. Specifically, young people who experience physiological manifestations of stress are more likely to be victims and those with behavioral manifestations are more likely to be aggressors. Based on these results, it is related that young people who experience situations of violence have higher levels of anxiety and depression, which in turn is linked to stress [21].

This work has a limitations. Some of the limitations of the present study include the lack of previous literature that directly addresses the relationship between healthy lifestyle in adolescence and its associations with stress, self-esteem, and violence roles. This lack of previous studies has made it difficult to contextualize and discuss the findings found in our work, limiting the ability to compare and contrast results or establish robust correlations. Furthermore, another limitation to consider is the disparity obtained in the figures reported between students who admit to belonging to one role of school violence (“victim/aggressor”) or the other (“non-victim/non-aggressor”). These data were obtained from the subjective perception of the students surveyed, which may have interfered with possible social desirability biases in the responses.

## 5. Conclusions

In conclusion, this research has allowed us to learn about the influence of a healthy lifestyle during adolescence in relation to stress, self-esteem, and school violence. Through the analysis of the data collected, relevant conclusions have been obtained that may have significant implications in the promotion of health and positive development of this population. An innovative and underexplored aspect in prior literature is the relationship between school violence roles, such as the bully and the victim, with healthy lifestyle habits, stress levels, and self-esteem during adolescence. This study emphasizes the importance of considering these roles within the context of a healthy lifestyle, as their influence on these variables can be significant but has generally been overlooked in previous research. This lack of exploration presents an opportunity for future investigations and the design of interventions addressing these less-examined yet potentially influential aspects on the psychological well-being of adolescents. Understanding how school violence roles intersect with a healthy lifestyle, stress, and self-esteem could open new avenues for effective strategies promoting health and emotional well-being in this critical developmental stage. One of the most prominent future lines of research is that interventions aimed at promoting a healthy lifestyle in adolescence could be designed and their impact on reducing stress, increasing self-esteem, and decreasing school violence could be evaluated. It is believed that the present study has provided valuable insights into the relationship between healthy lifestyle and aspects related to the psychological well-being of adolescents. However, further research is essential to gain a deeper understanding of these issues and thus contribute to the development of effective strategies to improve the quality of life of adolescents worldwide.

## Figures and Tables

**Table 1:** Descriptive and correlation matrix between healthy lifestyle, stress, and self-esteem (*N* = 743).

		(1)	(2)	(3)	(4)	(5)	(6)	(7)	(8)	(9)	(10)	(11)	(12)
Healthy Lifestyle	(1) Healthy diet	-											
	(2) Respecting meal times	0.44 ***	-										
	(3) Resting habits	0.38 ***	0.47 ***	-									
	(4) Tobacco use	−0.13 ***	−0.14 ***	−0.13 ***	-								
	(5) Alcohol consumption	−0.18 ***	−0.20 ***	−0.21 ***	0.58 ***	-							
	(6) Use of other drugs	−0.15 ***	−0.19 ***	−0.21 ***	0.68 ***	0.63 ***	-						
	(7) Physical activity	0.35 ***	0.28 ***	0.22 ***	−0.01	−0.02	−0.02	-					
Stress	(8) Emotional manifestations	−0.20 ***	−0.30 ***	−0.37 ***	0.14 ***	0.16 ***	0.22 ***	−0.20 ***	-				
	(9) Physiological manifestations	−0.15 ***	−0.29 ***	−0.29 ***	0.16 ***	0.19 ***	0.23 ***	−0.13 ***	0.69 ***	-			
	(10) Behavioral manifestations	−0.16 ***	−0.22 ***	−0.27 ***	0.26 ***	0.31 ***	0.36 ***	−0.07 *	0.51 ***	0.54 ***	-		
	(11) Total stress	−0.21 ***	−0.33 ***	−0.37 ***	0.20 ***	0.23 ***	0.29 ***	−0.17 ***	0.93 **	0.85 ***	0.72 ***	-	
Self-esteem	(12) Total self-esteem	0.23 ***	0.30 ***	0.35 ***	−0.09 *	−0.11 ***	−0.14 ***	0.23 ***	−0.59 ***	−0.45 ***	−0.32 ***	−0.57 ***	-
	Mean	9.32	9.90	9.46	4.40	8.28	7.72	17.19	30.88	13.50	12.18	56.55	28.58
	*SD*	2.65	3.16	3.16	2.81	4.32	4.15	5.10	8.61	4.54	3.71	14.65	5.91
	Min.	3	3	3	3	5	5	5	11	6	6	23	11
	Max.	15	15	15	15	24	25	25	55	29	30	103	40

Descriptive and correlation analysis; *SD* = standard deviation; Min = minimum; Max = maximum; *** *p* < 0.001; ** *p* < 0.01; * *p* < 0.05.

**Table 2:** Differences according to the profile of violence.

		**Victim**				***t***	***p***	***d***
		**Yes (*n* = 97)**		**No (*n* = 635)**				
		***Mean***	***SD***	***Mean***	***SD***			
Healthy lifestyle	Healthy diet	8.82	2.71	9.38	2.64	1.91	0.056	-
	Respecting meal times	9.37	3.33	10.01	3.13	1.85	0.064	-
	Rest habits	8.42	2.98	9.62	3.16	3.50 ***	<0.001	0.38
	Tobacco use	4.53	3.01	4.38	2.79	−0.48	0.630	-
	Alcohol consumption	9.01	4.85	8.20	4.25	−1.72	0.085	-
	Consumption of other drugs	8.54	4.31	7.60	4.13	−2.07 *	0.039	0.23
	Physical activity	16.24	4.95	17.35	5.11	2.00 *	0.046	0.22
Stress	Emotional Manifestations	33.87	8.15	30.42	8.64	−3.69 ***	<0.001	0.40
	Physiological Manifestations	15.44	4.63	13.18	4.47	−4.47 **	0.001	0.49
	Behavioral Manifestations	13.40	4.06	11.95	3.62	−3.62 ***	<0.001	0.40
	Total Stress	62.71	14.14	55.55	14.59	−4.52 ***	<0.001	0.49
Self-esteem	Total Self-esteem	26.68	5.95	28.89	5.86	3.44 ***	<0.001	0.38
		**Aggressor**				***t***	***p***	***d***
		**Yes (*n=* 57)**		**No (*n=* 676)**				
		***Mean***	***SD***	***Mean***	***SD***			
Healthy lifestyle	Healthy diet	8.29	2.54	9.40	2.63	3.16 **	0.002	0.44
	Respecting meal times	8.82	3.21	10.01	3.15	2.71 **	0.002	0.37
	Rest habits	8.16	3.50	9.57	3.10	3.25 **	0.009	0.45
	Tobacco use	5.23	3.54	4.34	2.75	−2.29 **	0.005	0.32
	Alcohol consumption	10.67	5.79	8.09	4.14	−4.35	0.069	-
	Consumption of other drugs	10.01	6.13	7.52	3.89	−4.38 **	0.002	0.60
	Physical activity	17.49	4.94	17.14	5.12	−0.49 **	0.004	0.07
Stress	Emotional Manifestations	31.71	6.97	30.78	8.76	−0.78	0.614	-
	Physiological Manifestations	14.00	4.84	13.44	4.50	−0.84	0.369	-
	Behavioral Manifestations	14.44	4.29	11.96	3.60	−4.90	0.401	-
	Total Stress	60.15	13.32	56.18	14.75	−1.96 ***	<0.001	0.27
Self-esteem	Total Self-esteem	27.59	4.87	28.66	5.98	1.31 *	0.036	0.18

Student’s *t*-test; *SD* = standard deviation; *** *p* < 0.001; ** *p* < 0.01; * *p* < 0.05.

**Table 3:** Gender differences between victims and perpetrators.

		**Victims**				***t***	***p***	***d***
		**Girls (*n* = 47)**		**Boys (*n* = 50)**				
		***Mean***	***SD***	***Mean***	***SD***			
Healthy lifestyle	Healthy diet	8.91	3.01	8.74	2.42	0.30	0.764	-
	Respecting meal times	9.00	3.21	9.71	3.43	−1.04	0.298	-
	Rest habits	8.86	2.90	8.00	3.02	1.43	0.155	-
	Tobacco use	4.38	2.89	4.67	3.15	−0.46	0.640	-
	Alcohol consumption	9.36	4.68	8.69	5.02	0.67	0.503	-
	Consumption of other drugs	8.14	3.67	8.91	4.84	−0.88	0.381	-
	Physical activity	14.78	4.51	17.61	5.00	−2.91 **	0.004	0.60
Stress	Emotional Manifestations	35.57	7.31	32.28	8.64	2.01 *	0.047	0.41
	Physiological Manifestations	15.86	4.46	15.04	4.80	0.87	0.386	-
	Behavioral Manifestations	12.23	3.17	14.50	4.50	−2.85 **	0.005	0.59
	Total Stress	63.65	12.93	61.83	15.27	0.63	0.528	-
Self-esteem	Total Self-esteem	25.05	5.14	28.22	6.29	−2.70 **	0.008	0.55
		**Aggressors**				***t***	***p***	***d***
		**Girls (*n* = 20)**		**Boys (*n* = 37)**				
		***Mean***	***SD***	***Mean***	***SD***			
Healthy lifestyle	Healthy diet	7.91	2.69	8.49	2.47	−0.82	0.413	-
	Respecting meal times	8.15	2.70	9.18	3.43	−1.16	0.250	-
	Rest habits	8.66	3.50	7.89	3.52	0.78	0.435	-
	Tobacco use	5.05	3.20	5.33	3.75	−0.28	0.779	-
	Alcohol consumption	10.71	5.59	10.65	5.97	0.03	0.970	-
	Consumption of other drugs	9.70	5.39	10.17	6.56	−0.27	0.783	-
	Physical activity	16.20	4.46	18.19	5.10	−1.46	0.149	-
Stress	Emotional Manifestations	32.96	5.82	31.04	7.51	0.99	0.327	-
	Physiological Manifestations	14.37	4.49	13.80	5.07	0.42	0.672	-
	Behavioral Manifestations	13.12	3.44	15.15	4.57	−1.74	0.087	-
	Total Stress	60.45	10.88	59.99	14.61	0.12	0.903	-
Self-esteem	Total Self-esteem	26.11	4.82	28.39	4.77	−1.71	0.094	-

Student’s *t*-test; *SD* = standard deviation; ** *p* < 0.01; * *p* < 0.05.

**Table 4:** Differences according to the different groups of self-esteem.

Scale		Resilience Groups	*N*	Mean	*SD*	ANOVA		Difference in Averages
						*F*	Sig.	
Healthy lifestyle	Healthy diet	Low (g1)	214	8.63	2.74	18.11	<0.001	|g1–g2| |g2–g3| *** |g2–g3| *** |g1–g3| *** |g1–g3| ***
		Mean (g2)	215	9.07	2.48			
		High (g3)	314	9.96	2.56			
	Respecting meal times	Low (g1)	214	8.81	3.31	28.10	<0.001	|g1–g2| * |g1–g2| * |g2–g3| *** |g2–g3| *** |g1–g3| *** |g1–g3| ***
		Mean (g2)	215	9.67	2.82			
		High (g3)	314	10.81	3.03			
	Rest habits	Low (g1)	214	8.10	2.98	37.14	<0.001	|g1–g2| *** |g1–g2| *** |g2–g3| *** |g2–g3| *** |g1–g3| *** |g1–g3| ***
		Mean (g2)	215	9.43	2.88			
		High (g3)	314	10.40	3.11			
	Tobacco use	Low (g1)	214	4.75	3.31	3.27	0.039	|g1–g2| |g2–g3| |g1–g3|
		Mean (g2)	215	4.46	2.57			
		High (g3)	314	4.12	2.57			
	Alcohol consumption	Low (g1)	214	8.98	4.59	6.49	0.002	|g1–g2| |g2–g3| |g1–g3| * |g1–g3| *
		Mean (g2)	215	8.49	4.16			
		High (g3)	314	7.65	4.16			
	Consumption of other drugs	Low (g1)	214	8.46	4.54	7.48	<0.001	|g1–g2| |g2–g3| |g1–g3| *** |g1–g3| ***
		Mean (g2)	215	7.90	4.04			
		High (g3)	314	7.08	3.85			
	Physical activity	Low (g1)	214	15.84	5.30	16.80	<0.001	|g1–g2| |g2–g3| ** |g1–g3| *** |g1–g3| ***
		Mean (g2)	215	16.84	4.55			
		High (g3)	314	18.35	5.08			
Stress	Emotional Manifestations	Low (g1)	214	37.07	8.02	145.17	<0.001	|g1–g2| *** |g1–g2| *** |g2–g3| ** |g1–g3| **
		Mean (g2)	215	31.69	6.88			
		High (g3)	314	26.10	7.08			
	Physiological Manifestations	Low (g1)	214	16.00	4.90	77.77	<0.001	|g1–g2| *** |g1–g2| *** |g2–g3| *** |g2–g3| *** |g1–g3| **
		Mean (g2)	215	13.96	4.07			
		High (g3)	314	11.48	3.56			
	Behavioral Manifestations	Low (g1)	214	13.47	3.98	36.31	<0.001	|g1–g2| |g2–g3| *** |g2–g3| *** |g1–g3| *** |g1–g3| ***
		Mean (g2)	215	12.72	3.56			
		High (g3)	314	10.92	3.20			
	P. Total Stress	Low (g1)	214	66.53	14.05	134.104	<0.001	|g1–g2| *** |g1–g2| *** |g2–g3| ** |g1–g3| **
		Mean (g2)	215	58.38	12.13			
		High (g3)	314	48.50	11.77			

ANOVA test; *SD* = standard deviation; *** *p* < 0.001; ** *p* < 0.01; * *p* < 0.05.

**Table 5:** Probability of being a victim and being a perpetrator.

**Victim**						
**Variables**	**ß**	**Standard Error**	**Sig.**	**Exp (ß)**	**95% C.I. for EXP (ß)**	
					**Inferior**	**Superior**
Healthy diet	−0.01	0.04	0.720	0.98	0.89	1.08
Respecting meal times	0.04	0.04	0.341	1.04	0.95	1.13
Rest habits	−0.07	0.04	0.094	0.93	0.85	1.01
Tobacco use	−0.07	0.05	0.176	0.92	0.82	1.03
Alcohol consumption	0.01	0.03	0.571	1.01	0.95	1.08
Consumption of other drugs	0.03	0.03	0.347	1.03	0.96	1.11
Physical activity	−0.02	0.02	0.341	0.97	0.93	1.02
Emotional Manifestations	−0.00	0.02	0.661	0.99	0.95	1.03
Physiological Manifestations	0.07	0.03	0.040	1.07	1.00	1.14
Behavioral Manifestations	0.03	0.03	0.359	1.03	0.96	1.10
Self-esteem	−0.02	0.02	0.301	0.97	0.93	1.02
Constant	−1.64	1.17	0.162	0.19		
**Aggressor**						
**Variables**	**ß**	**Standard Error**	**Sig.**	**Exp (ß)**	**95% C.I. for EXP (ß)**	
					**Inferior**	**Superior**
Healthy diet	−0.11	0.06	0.086	0.89	0.78	1.01
Respecting meal times	−0.04	0.05	0.421	0.95	0.85	1.06
Rest habits	−0.05	0.05	0.321	0.94	0.85	1.05
Tobacco use	−0.08	0.06	0.188	0.91	0.80	1.04
Alcohol consumption	0.05	0.03	0.146	1.05	0.98	1.13
Consumption of other drugs	0.06	0.04	0.122	1.06	0.98	1.16
Physical activity	0.04	0.03	0.128	1.04	0.98	1.11
Emotional Manifestations	−0.03	0.02	0.191	0.96	0.91	1.01
Physiological Manifestations	−0.05	0.04	0.182	0.94	0.86	1.02
Behavioral Manifestations	0.15	0.04	<0.001	1.16	1.07	1.26
Self-esteem	−0.02	0.03	0.375	0.97	0.91	1.03
Constant	−1.45	1.47	0.324	0.23		

Binary logistic regression.

## Data Availability

The data presented in this study are available on request from the corresponding author.

## References

[1] Organización Mundial de la Salud (OMS) (1986). Estilo de Vida.

[2] Organización Mundial de la Salud (OMS) (2023). Salud del Adolescente.

[3] Viner R.M., Ross D., Hardy R., Kuh D., Power C., Johnson A., Wellings K., McCambridge J., Cole T.J., Kelly Y. (2015). Life Course Epidemiology: Recognising the Importance of Adolescence. J. Epidemiol. Community Health.

[4] Mastorci F., Bastiani L., Doveri C., Trivellini G., Casu A., Vassalle C., Pingitore A. (2020). Adolescent Health: A Framework for Developing an Innovative Personalized Well-Being Index. Front. Pediatr..

[5] Anderson E., Durstine J.L. (2019). Physical Activity, Exercise, and Chronic Diseases: A Brief Review. Sports Med. Health Sci..

[6] Browne J., Becker D., Orellana L., Ryan J., Walker T., Whelan J., Alston L., Egan M., Johnson B., Rossignoli A. (2022). Healthy Weight, Health Behaviours and Quality of Life among Aboriginal Children Living in Regional Victoria. Aust. N. Z. J. Public Health.

[7] Rodriguez-Ayllon M., Cadenas-Sánchez C., Estévez-López F., Muñoz N.E., Mora-Gonzalez J., Migueles J.H., Molina-García P., Henriksson H., Mena-Molina A., Martínez-Vizcaíno V. (2019). Role of Physical Activity and Sedentary Behavior in the Mental Health of Preschoolers, Children and Adolescents: A Systematic Review and Meta-Analysis. Sports Med..

[8] Menakaya N.C., Menakaya I.N. (2022). Qualitative Study Exploring Perceptions, Attitudes and Practices of Adolescent University Students in Lagos, Nigeria, towards a Healthy Lifestyle. Afr. J. Prim. Health Care Fam. Med..

[9] Bektas İ., Kudubeş A.A., Ayar D., Bektas M. (2021). Predicting the Healthy Lifestyle Behaviors of Turkish Adolescents Based on Their Health Literacy and Self-Efficacy Levels. J. Pediatr. Nurs..

[10] Marques A., Loureiro N., Avelar-Rosa B., Naia A., de Matos M.G. (2020). Adolescents’ Healthy Lifestyle. J. Pediatr..

[11] Haines J., Haycraft E., Lytle L., Nicklaus S., Kok F.J., Merdji M., Fisberg M., Moreno L.A., Goulet O., Hughes S.O. (2019). Nurturing Children’s Healthy Eating: Position Statement. Appetite.

[12] Lu C., Chi X., Liang K., Chen S.-T., Huang L., Guo T., Jiao C., Yu Q., Veronese N., Soares F.C. (2020). Moving More and Sitting Less as Healthy Lifestyle Behaviors Are Protective Factors for Insomnia, Depression, and Anxiety Among Adolescents During the COVID-19 Pandemic. Psychol. Res. Behav. Manag..

[13] Waterlander W.E., Luna Pinzon A., Verhoeff A., den Hertog K., Altenburg T., Dijkstra C., Halberstadt J., Hermans R., Renders C., Seidell J. (2020). A System Dynamics and Participatory Action Research Approach to Promote Healthy Living and a Healthy Weight among 10–14-Year-Old Adolescents in Amsterdam: The LIKE Programme. Int. J. Environ. Res. Public Health.

[14] Lindholdt L., Labriola M., Andersen J.H., Kjeldsen M.-M.Z., Obel C., Lund T. (2022). Perceived Stress among Adolescents as a Marker for Future Mental Disorders: A Prospective Cohort Study. Scand. J. Public Health.

[15] Darling K.E., Ruzicka E.B., Fahrenkamp A.J., Sato A.F. (2019). Perceived Stress and Obesity-Promoting Eating Behaviors in Adolescence: The Role of Parent-Adolescent Conflict. Fam. Syst. Health.

[16] Kappes C., Stein R., Körner A., Merkenschlager A., Kiess W. (2023). Stress, Stress Reduction and Obesity in Childhood and Adolescence. Horm. Res. Paediatr..

[17] Milas G., Klarić I.M., Malnar A., Šupe-Domić D., Slavich G.M. (2019). Socioeconomic Status, Social-cultural Values, Life Stress, and Health Behaviors in a National Sample of Adolescents. Stress Health.

[18] Oksanen A., Miller B.L., Savolainen I., Sirola A., Demant J., Kaakinen M., Zych I. (2020). Social Media and Access to Drugs Online: A Nationwide Study in the United States and Spain among Adolescents and Young Adults. Eur. J. Psychol. Appl. Leg. Context.

[19] Koopmann A., Georgiadou E., Reinhard I., Müller A., Lemenager T., Kiefer F., Hillemacher T. (2021). The Effects of the Lockdown during the COVID-19 Pandemic on Alcohol and Tobacco Consumption Behavior in Germany. Eur. Addict. Res..

[20] Pérez-Fuentes M., Molero Jurado M., Barragán Martín A., Gázquez Linares J. (2019). Family Functioning, Emotional Intelligence, and Values: Analysis of the Relationship with Aggressive Behavior in Adolescents. Int. J. Environ. Res. Public Health.

[21] Molero M.M., Martos Á., Barragán A.B., Pérez-Fuentes M.C., Gázquez J.J. (2022). Anxiety and Depression from Cybervictimization in Adolescents: A Metaanalysis and Meta-Regression Study. Eur. J. Psychol. Appl. Leg. Context.

[22] Wright L.J., Veldhuijzen van Zanten J.J.C.S., Williams S.E. (2023). Examining the Associations between Physical Activity, Self-esteem, Perceived Stress, and Internalizing Symptoms among Older Adolescents. J. Adolesc..

[23] Mikkelsen H.T., Haraldstad K., Helseth S., Skarstein S., Småstuen M.C., Rohde G. (2020). Health-Related Quality of Life Is Strongly Associated with Self-Efficacy, Self-Esteem, Loneliness, and Stress in 14–15-Year-Old Adolescents: A Cross-Sectional Study. Health Qual. Life Outcomes.

[24] Rosenberg M. (1979). Conceiving the Self.

[25] Poudel A., Gurung B., Khanal G.P. (2020). Perceived Social Support and Psychological Wellbeing among Nepalese Adolescents: The Mediating Role of Self-Esteem. BMC Psychol..

[26] Jafflin K., Pfeiffer C., Bergman M.M. (2019). Effects of Self-Esteem and Stress on Self-Assessed Health: A Swiss Study from Adolescence to Early Adulthood. Qual. Life Res..

[27] Liu E., Chang S.-H. (2022). Self-Esteem and Weight Status of Young Adults: Findings from a Pilot Study. J. Educ. Health Promot..

[28] Sampasa-Kanyinga H., Lien A., Hamilton H.A., Chaput J.-P. (2022). Canadian 24-h Movement Guidelines, Life Stress, and Self-Esteem Among Adolescents. Front. Public Health.

[29] Jaskulska S., Jankowiak B., Pérez-Martínez V., Pyżalski J., Sanz-Barbero B., Bowes N., De Claire K., Neves S., Topa J., Silva E. (2022). Bullying and Cyberbullying Victimization and Associated Factors among Adolescents in Six European Countries. Sustainability.

[30] Hsieh H.-F., Mistry R., Lee D.B., Scott B.A., Eisman A.B., Heinze J.E., Zimmerman M.A. (2021). The Longitudinal Association Between Exposure to Violence and Patterns of Health Risk Behaviors Among African American Youth. Am. J. Health Promot..

[31] Oberth C., Goulter N., McMahon R.J. (2022). The Comparative and Cumulative Impact of Different Forms of Violence Exposure during Childhood and Adolescence on Long-Term Adult Outcomes. Dev. Psychopathol..

[32] Varela J.J., Guzmán J., Alfaro J., Reyes F. (2019). Bullying, Cyberbullying, Student Life Satisfaction and the Community of Chilean Adolescents. Appl. Res. Qual. Life.

[33] Molero M.M., Pérez-Fuentes M.C., Martos Á., Pino R.M., Gázquez J.J. (2023). Network Analysis of Emotional Symptoms and Their Relationship with Different Types of Cybervictimization. Eur. J. Psychol. Appl. Leg. Context.

[34] Shah N., Rao S., Inam S., Jawed N., Siiger C., Adil S.O., Shafique K. (2019). Healthy Lifestyle as a Preventive Measure against Victimization among School-Going Adolescents. East. Mediterr. Health J..

[35] Vandenbroucke J.P., von Elm E., Altman D.G., Gøtzsche P.C., Mulrow C.D., Pocock S.J., Poole C., Schlesselman J.J., Egger M. (2007). Strengthening the Reporting of Observational Studies in Epidemiology (STROBE). Epidemiology.

[36] Leyton-Román M., Mesquita S., Jiménez-Castuera R. (2021). Validation of the Spanish Healthy Lifestyle Questionnaire. Int. J. Clin. Health Psychol..

[37] Escobar Espejo M., Blanca M.J., Fernández-Baena F.J., Trianes-Torres M.V. (2011). Adaptación Española de La Escala de Manifestaciones de Estrés Del Student Stress Inventory (SSI-SM). Psicothema.

[38] Fimian M.J., Fastenau P.A., Tashner J.H., Cross A.H. (1989). The Measure of Classroom Stress and Burnout among Gifted and Talented Students. Psychol. Sch..

[39] Atienza F.L., Moreno Y., Balaguer I. (2000). Análisis de La Dimensionalidad de La Escala de Autoestima de Rosenberg En Una Muestra de Adolescentes Valencianos. Rev. Psicol. Universitas Tarraconensias.

[40] Rosenberg M. (1965). Society and the Adolescent Self-Image.

[41] IBM Corp (2021). IBM SPSS Statistics for Macintosh.

[42] Cronbach L.J. (1951). Coefficient Alpha and the Internal Structure of Tests. Psychometrika.

[43] Pearson K.X. (1900). On the Criterion That a given System of Deviations from the Probable in the Case of a Correlated System of Variables Is Such That It Can Be Reasonably Supposed to Have Arisen from Random Sampling. Lond. Edinb. Dublin Philos. Mag. J. Sci..

[44] Cohen J. (1988). Statistical Power Analysis for the Behavioral Sciences.

[45] Cohen J. (1992). A Power Primer. Psychol. Bull..

[46] Field A., Miles J., Field Z. (2012). Discovering Statistics Using R.

